# Ketogenic Diet Regulates Cardiac Remodeling and Calcium Homeostasis in Diabetic Rat Cardiomyopathy

**DOI:** 10.3390/ijms242216142

**Published:** 2023-11-09

**Authors:** Ting-I Lee, Nguyen Ngoc Trang, Ting-Wei Lee, Satoshi Higa, Yu-Hsun Kao, Yao-Chang Chen, Yi-Jen Chen

**Affiliations:** 1 Division of Endocrinology and Metabolism, Department of Internal Medicine, School of Medicine, College of Medicine, Taipei Medical University, Taipei 11031, Taiwan; agleems29@gmail.com (T.-I.L.); b8801138@tmu.edu.tw (T.-W.L.); 2 Division of Endocrinology and Metabolism, Department of Internal Medicine, Wan Fang Hospital, Taipei Medical University, Taipei 11696, Taiwan; 3 Radiology Center, Bach Mai Hospital, Hanoi 100000, Vietnam; drnguyenngoctrang@gmail.com; 4 Cardiac Electrophysiology and Pacing Laboratory, Division of Cardiovascular Medicine, Makiminato Central Hospital, Makiminato Urasoe City, Okinawa 901-2131, Japan; sa_higa@yahoo.co.jp; 5 Graduate Institute of Clinical Medicine, College of Medicine, Taipei Medical University, Taipei 11031, Taiwan; yuhsunkao@gmail.com; 6 Department of Medical Education and Research, Wan Fang Hospital, Taipei Medical University, Taipei 11696, Taiwan; 7 Department of Biomedical Engineering, National Defense Medical Center, Taipei 11490, Taiwan; 8 Cardiovascular Research Center, Wan Fang Hospital, Taipei Medical University, Taipei 11696, Taiwan

**Keywords:** arrhythmias, calcium homeostasis, diabetic cardiomyopathy, electrophysiology, ketogenic diet

## Abstract

A ketogenic diet (KD) might alleviate patients with diabetic cardiomyopathy. However, the underlying mechanism remains unclear. Myocardial function and arrhythmogenesis are closely linked to calcium (Ca^2+^) homeostasis. We investigated the effects of a KD on Ca^2+^ homeostasis and electrophysiology in diabetic cardiomyopathy. Male Wistar rats were created to have diabetes mellitus (DM) using streptozotocin (65 mg/kg, intraperitoneally), and subsequently treated for 6 weeks with either a normal diet (ND) or a KD. Our electrophysiological and Western blot analyses assessed myocardial Ca^2+^ homeostasis in ventricular preparations in vivo. Unlike those on the KD, DM rats treated with an ND exhibited a prolonged QTc interval and action potential duration. Compared to the control and DM rats on the KD, DM rats treated with an ND also showed lower intracellular Ca^2+^ transients, sarcoplasmic reticular Ca^2+^ content, sodium (Na^+^)-Ca^2+^ exchanger currents (reverse mode), L-type Ca^2+^ contents, sarcoplasmic reticulum ATPase contents, Cav1.2 contents. Furthermore, these rats exhibited elevated ratios of phosphorylated to total proteins across multiple Ca^2+^ handling proteins, including ryanodine receptor 2 (RyR2) at serine 2808, phospholamban (PLB)-Ser16, and calmodulin-dependent protein kinase II (CaMKII). Additionally, DM rats treated with an ND demonstrated a higher frequency and incidence of Ca^2+^ leak, cytosolic reactive oxygen species, Na^+^/hydrogen-exchanger currents, and late Na^+^ currents than the control and DM rats on the KD. KD treatment may attenuate the effects of DM-dysregulated Na^+^ and Ca^2+^ homeostasis, contributing to its cardioprotection in DM.

## 1. Introduction

Low-carbohydrate diets have gained popularity recently because of the increased awareness regarding the harmful metabolic effects of processed carbohydrates. The ketogenic diet (KD) is a high-fat, low-carbohydrate diet that serves around 60% of fats for daily energy needs. It has been linked to mitigating epileptic seizures in children [1] and several other neurological conditions [2]. When dietary fat replaces carbohydrates in the liver, fat will be transformed into ketones, including beta-hydroxybutyrate (BHB), acetoacetate, acetone, and fatty acids (FAs). Evidence of the KD’s possible health advantages includes neurological illnesses, cancer, and mitochondrial diseases [3,4,5], as well as BHB improved endogenous antioxidant defense and mitochondrial performance [5]. Moreover, increased circulating ketone levels in patients with heart failure may also improve their cardiac health [4,6] because, as oxygen-efficient, highly energetic fuel, ketone bodies may enhance the work capacity of the myocardium at risk [7,8].

Diabetic cardiomyopathy is clinically asymptomatic in its early stage. It is marked by increased fibrosis, reduced early diastolic filling, elevated atrial filling, and enlargement, and raised left ventricular (LV) end-diastolic pressure [9]. The second stage involves cardiac remodeling, LV hypertrophy, and advanced diastolic dysfunction, eventually leading to clinical heart failure with normal ejection fraction (EF) [10]. As diabetic cardiomyopathy progresses, diastolic dysfunction with decreased cardiac compliance may coevolve with systolic dysfunction, resulting in reduced EF [10]. Therefore, patients with diabetes mellitus (DM) can exhibit a spectrum of cardiomyopathy, ranging from subclinical cardiac abnormalities to diastolic and then systolic dysfunction, cumulating in reduced EF [11]. The pathological impact of diabetic cardiomyopathy in terms of its underlying mechanism and functional implications warrants clarification; as such, the optimal therapeutic strategy for diabetic cardiomyopathy remains unknown.

Moreover, it remains unclear whether KD will modulate sodium (Na^+^) or calcium (Ca^2+^) homeostasis through its potential consequences on the myocardial electrical and structural remodeling in DM hearts. Our earlier investigation demonstrated that sodium-glucose co-transporter-2 inhibitor (SLGT2i) modulated Na^+^ or Ca^2+^ homeostasis by decreasing oxidative stress in diabetic cardiomyopathy [12]. In the cardiomyocytes, SLGT2i may increase energy utilization from the ketone bodies, thereby improving the myocardial function with an antiarrhythmic potential [13]. Furthermore, we recently illustrated that empagliflozin regulates glucose and FA metabolism [14]. Consequently, we hypothesize that a KD may modulate the electrical and structural remodeling of the heart and alter the Na^+^ or Ca^2+^ homeostasis in diabetic cardiomyopathy.

## 2. Results

### 2.1. Myocardial Function and Electrophysiology of the Ventricular Myocytes of Control Rats, DM Rats on a Normal Diet (ND), and DM Rats on a KD

**Figure 1:**
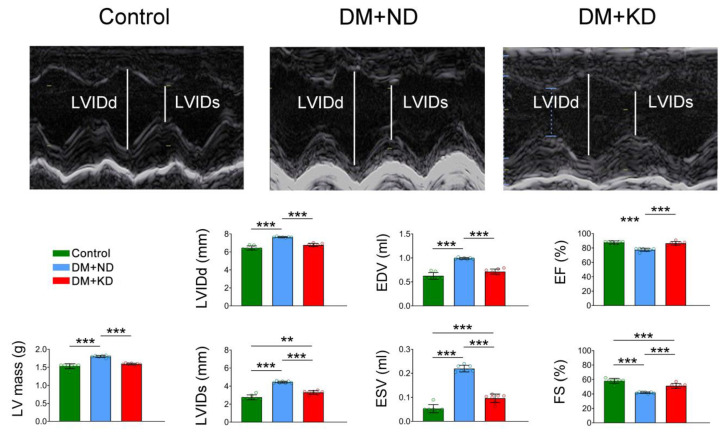
Effects of a ketogenic diet (KD) on echocardiograms in the control rats (control), diabetes mellitus (DM) rats on a normal diet (DM + ND), and DM rats on the KD (DM + KD). Average data of the left ventricular internal dimension at end-diastole (LVIDd), left ventricular internal dimension at end-systole (LVIDs), end-diastolic volume (EDV), end-systolic volume (ESV), ejection fraction (EF), fractional shortening (FS), and left ventricular (LV) mass. Number of rats (N) = 7 per group. Statistical significance was assessed using a one-way analysis of variance (ANOVA) with Tukey’s honest significant difference (HSD) test. ** *p* < 0.01; *** *p* < 0.005.

Figure 1 represents the echocardiograms of the control, DM rats on an ND, and DM rats on the KD. The DM rats on an ND revealed elevated values of left ventricular internal dimension at end-diastole (LVIDd), left ventricular internal dimension at end-systole (LVIDs), end-diastole volume (EDV), end-systole volume (ESV), and LV mass in comparison to either the control rats or DM rats on the KD. The EF and fractional shortening (FS) values were lower in the DM rats on an ND than in the control and DM rats on the KD. Similar to our earlier studies [15], the current DM rats on an ND showed a higher heart-to-body weight ratio than the control and DM rats on the KD (Supplementary Table S1).

**Figure 2:**
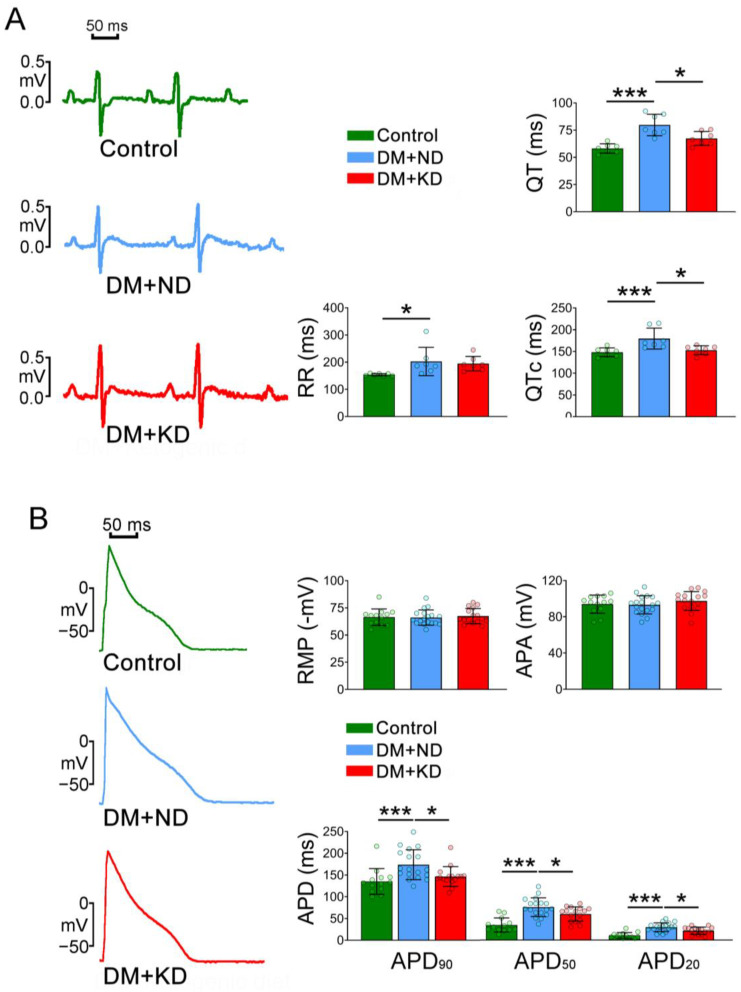
Changes in the electrocardiogram and action potentials (Aps) of ventricular myocytes in the control rats (control), diabetes mellitus (DM) rats on a normal diet (DM + ND), and DM rats on the ketogenic diet (DM + KD). (**A**) Average data and representative tracings of the electrocardiograms before and after treatment in ventricular myocytes of the control (N = 7), DM + ND (N = 7), and DM + KD (N = 7) rats. (**B**) Average data and representative AP tracings in the ventricular myocytes of the control (n = 13), DM + ND (n = 18), and DM + KD (n = 16). Abbreviations: QT: QT interval, RR: RR interval, QTc: corrected QT interval, N: number of rats, n: number of cardiomyocytes isolated from the rats, RMP: resting membrane potential, APA: action potential amplitude, APD_20_, APD_50_, and APD_90_: action potential durations of repolarization at 20%, 50%, and 90%, respectively, number of rats (N) = 7 per group, n: number of cardiomyocytes isolated from the rats. Statistical significance was assessed using a one-way analysis of variance (ANOVA) with Tukey’s honest significant difference (HSD) test. * *p* < 0.05; *** *p* < 0.005.

DM rats on an ND had longer QT intervals (80 ± 9.8 ms) and corrected QT (QTc) intervals (180 ± 24.1 ms) than either the control rats (58 ± 4.3 and 148 ± 10.1 ms, respectively; Tukey’s honest significance test (HSD) test, both *p* < 0.005) or DM rats on the KD (67 ± 6.4 and 153 ± 10.3, respectively; Tukey’s HSD test, *p* < 0.01 and Tukey’s HSD test, *p* < 0.05, respectively; Figure 2A). The RR intervals were longer in DM rats on an ND than in the control rats (203 ± 51.9 vs. 154 ± 4.2 ms; Tukey’s HSD test, *p* < 0.05), but the RR intervals between DM rats on an ND and the KD (203 ± 51.9 vs. 195 ± 26.5 ms; Tukey’s HSD test, *p* < 0.9) were similar (Figure 2A).

In the ventricular myocytes of DM rats on an ND, action potential durations (APDs) at 20% (APD_20_), 50% (APD_50_), and 90% (APD_90_) APD_90_ were 29.6 ± 10.1, 75.7 ± 21.5, and 173.7 ± 34.4 ms, respectively (n = 18); they were longer than those in the ventricular myocytes of the control rats (10.8 ± 6.6, 34.7 ± 16.2, and 135.2 ± 29.6 ms, respectively; n = 13; Tukey’s HSD test, *p* < 0.005) and of DM rats on the KD (21.5 ± 8.3, 59.9 ± 16.4, and 146.5 ± 22.7 ms, respectively; n = 16; Tukey’s HSD test, *p* < 0.05; Figure 2B). Compared with the control rats, DM rats on the KD had ventricular myocytes with longer APD_20_ and APD_50_ (Tukey’s HSD test, *p* < 0.005) but with similar APD_90_. Resting membrane potential (RMP) and action potential amplitude (APA) were similar in the control, DM rats on ND, and DM rats on KD groups (Figure 2B).

### 2.2. Ca^2+^ Stores in the Ventricular Myocytes of DM with or without a KD

**Figure 3:**
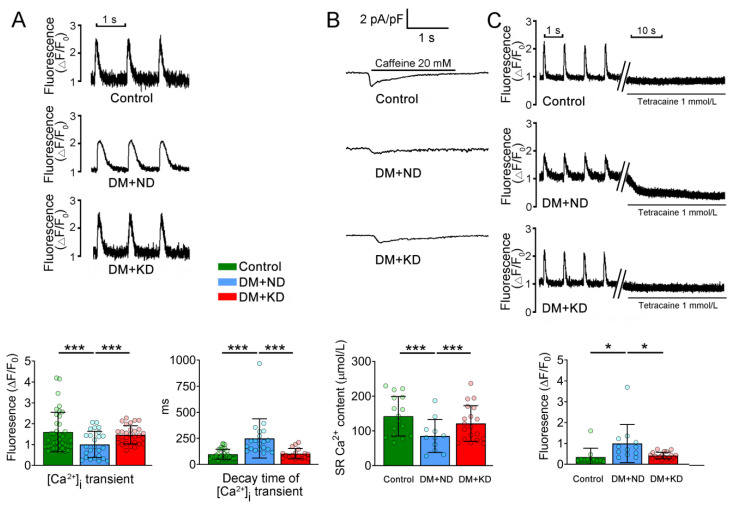
[Ca^2+^]_i_ transients, Ca^2+^ stores measured from caffeine (20 mM)-induced Ca^2+^ transients, and Ca^2+^ leak in the ventricular myocytes of the control rats (control), diabetes mellitus (DM) rats on a normal diet (DM + ND), and DM rats on the ketogenic diet (DM + KD). (**A**) Average data and representative tracings of [Ca^2+^]_i_ transients and decay time of [Ca^2+^]_i_ transients in the ventricular myocytes of the control (n = 37), DM + ND (n = 25), and DM + KD (n = 29) rats. (**B**) Average data and representative tracings of Ca^2+^ stores in the ventricular myocytes of the control (n = 31), DM + ND (n = 20), and DM + KD (n = 17) rats. (**C**) Average data and representative tracing of the incidence and frequency of Ca^2+^ leak in the ventricular myocytes of the control (n = 11), DM + ND (n = 12), and DM + KD (n = 19) rats. Abbreviations: [Ca^2+^]_i_: intracellular calcium, Ca^2+^:calcium, n: number of cardiomyocytes isolated from the rats. Statistical significance was assessed using a one-way analysis of variance (ANOVA) with Tukey’s honest significant difference (HSD) test. * *p* < 0.05; *** *p* < 0.005.

Next, we assessed Ca^2+^ homeostasis in the ventricular myocytes of the control rats, DM rats on an ND, and DM rats on the KD, intracellular Ca^2+^ ([Ca^2+^]_i_) transients were 55% and 45% fewer in DM rats on ND (1.0 ± 0.6 F/F_0_, n = 25) than in the control rats (1.6 ± 0.9 F/F_0_, n = 37) and DM rats on the KD (1.5 ± 0.4 F/F_0_, n = 29), respectively (Tukey’s HSD test, both *p* < 0.005). However, the control and DM rats on the KD had the same [Ca^2+^]_i_ transients (Figure 3). Additionally, the decay time of [Ca^2+^]_i_ transients in the ventricular myocytes of DM rats on an ND was significantly longer (249.6 ± 188.1 ms, n = 20) than that in the ventricular myocytes of the control rats (97.5 ± 47.7 ms, n = 31) and DM rats on the KD (103.2 ± 49.5 ms, n = 17; Tukey’s HSD test: both *p* < 0.005). Nevertheless, the control and DM rats on KD ventricular myocytes had similar decay times of [Ca^2+^]_i_ transients (Figure 3).

As illustrated in Figure 3, [Ca^2+^]_i_ leak in the ventricular myocytes of DM rats on an ND was significantly increased (1 ± 0.9 F/F_0_, n = 12) compared with that in the ventricular myocytes of the control rats (0.3 ± 0.4 F/F_0_, n = 11) and of DM rats on the KD (0.4 ± 0.2 F/F_0_, n = 19; Tukey’s HSD test: both *p* < 0.05). By contrast, the control and DM rats on the KD ventricular myocytes had similar [Ca^2+^]_i_ leak (Figure 3).

### 2.3. Effects of KD L-Type Ca^2+^ Current, (I_Ca-L_) Current, and Na^+^/Ca^2+^ Exchanger (NCX) Current in the Ventricular Myocytes of DM Rats with and without KD

**Figure 4:**
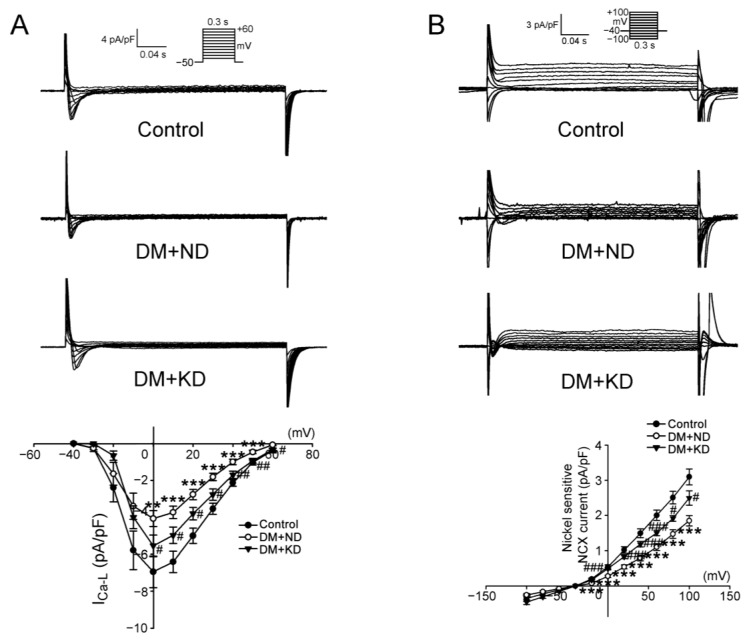
I_Ca-L_ and NCX current in the ventricular myocytes of the control rats (control), diabetes mellitus (DM) rats on a normal diet (DM + ND), and DM rats on the ketogenic diet (DM + KD). (**A**) Representative tracings of I_Ca-L_ current and the current–voltage relationship of I_Ca-L_ in the ventricular myocytes of the control (n = 16), DM + ND (n = 14), and DM + KD (n = 13) rats. (**B**) Representative tracings of NCX current and the current–voltage relationship of NCX current in the ventricular myocytes of the control (n = 12), DM + ND (n = 21), and DM + KD (n = 12) rats. Abbreviations: I_Ca-L_: L-type calcium current, NCX: sodium/calcium exchanger, n: number of cardiomyocytes isolated from the rats. Statistical significance was assessed using a one-way analysis of variance (ANOVA) with Tukey’s honest significant difference (HSD) test. ^#^ *p* < 0.05 vs. the controls; ^##^ *p* < 0.01 vs. the controls; ^###^ *p* < 0.005 vs. the controls; ** *p* < 0.01 vs. DM + KD rats; *** *p* < 0.005 vs. DM + KD rats.

I_Ca-L_ density (in the peak *I*_Ca-L_ elicited from −50 to 10 mV) in the ventricular myocytes of DM rats on an ND was higher (3.7 ± 2.8 pA/pF, n = 17) than that in the control rats (6.4 ± 2.3 pA/pF, n = 16; Tukey’s HSD test, *p* < 0.005) and DM rats on the KD (5.0 ± 1.6 pA/pF, n = 13; Tukey’s HSD test, *p* < 0.05; Figure 4A). Figure 4B illustrates the tracings and current-voltage relationships of nickel-sensitive NCX currents in the ventricular myocytes of the control rats, DM rats on an ND, and DM rats on the KD. We noted a smaller reverse mode of nickel-sensitive NCX currents in the ventricular myocytes of DM rats on an ND (0.28 ± 0.2 pA/pF, n = 21) than in the control rats (0.55 ± 0.2 pA/pF, n = 12) and of DM rats on the KD (0.49 ± 0.1 pA/pF, n = 12; Tukey’s HSD test, both *p* < 0.005).

### 2.4. Effects of KD on Late Sodium Current (I_Na-Late_) and Sodium/Hydrogen (Na^+^/H^+^) Exchanger Current on the Ventricular Myocytes DM Rats with or without a KD

**Figure 5:**
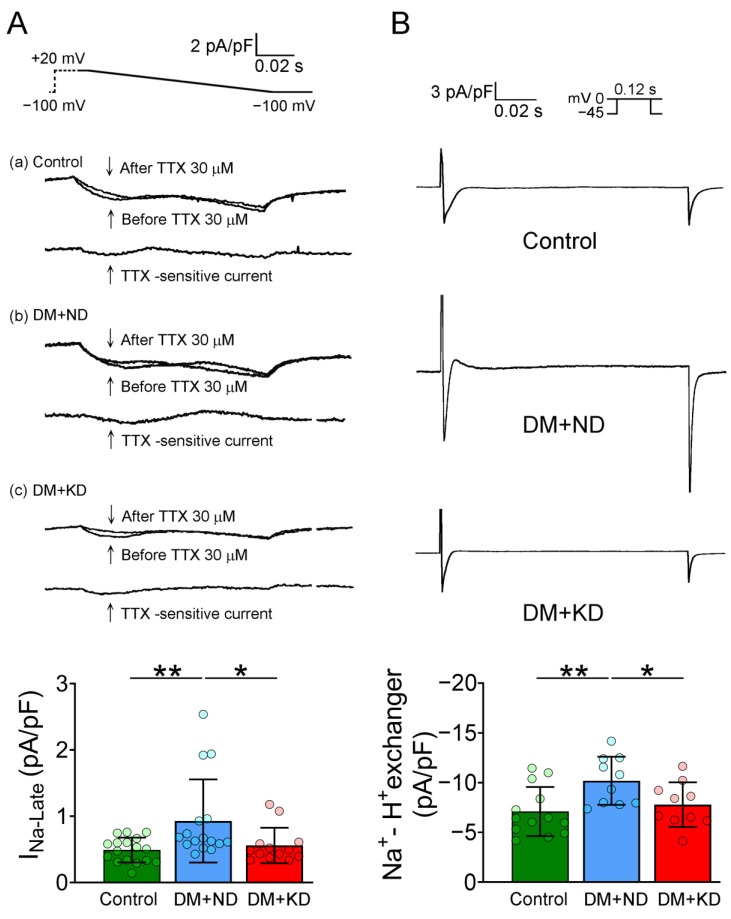
I_Na-Late_ and Na^+^/H^+^ exchanger current in the ventricular myocytes of the control rats (control), diabetes mellitus (DM) rats on a normal diet (DM + ND), and DM rats on the ketogenic diet (DM + KD). (**A**) Average data and representative tracing of I_Na-Late_ current in the ventricular myocytes of the (a) control (n = 17), (b) DM + ND (n = 16), and (c) DM + KD (n = 13) rats. (**B**) Average data and representative tracings of Na^+^/H^+^ exchanger current in the ventricular myocytes of the control (n = 11), DM + ND (n = 14), and DM + KD (n = 14) rats. Abbreviations: I_Na-Late_: late sodium current, Na^+^/H^+^ exchanger: sodium/hydrogen exchanger, n: number of cardiomyocytes isolated from the rats. Statistical significance was assessed using a one-way analysis of variance (ANOVA) with Tukey’s honest significant difference (HSD) test. ** *p* < 0.01 vs. control; * *p* < 0.05 vs. DM + KD.

I_Na-late_ density of DM rats on ND ventricular myocytes was higher (0.92 ± 0.6 pA/pF, n = 17) than that in the ventricular myocytes of the control rats (0.49 ± 0.2 pA/pF, n = 16; Tukey’s HSD test: *p* < 0.01) and of DM rats on the KD (0.56 ± 0.3 pA/pF, n = 13; Tukey’s HSD test, *p* < 0.05; Figure 5). Na^+^/H^+^ exchanger current density of DM rats on ND ventricular myocytes was more significant (10.2 ± 2.6 pA/pF, n = 16) than that in the ventricular myocytes of the control rats (7.1 ± 2.5 pA/pF, n = 17) and of DM rats on the KD (7.8 ± 2.2 pA/pF, n = 13; Tukey’s HSD test, both *p* < 0.05; Figure 5).

### 2.5. Oxidative Stress of the Ventricular Myocytes of the Control Rats and DM with or without a KD

**Figure 6:**
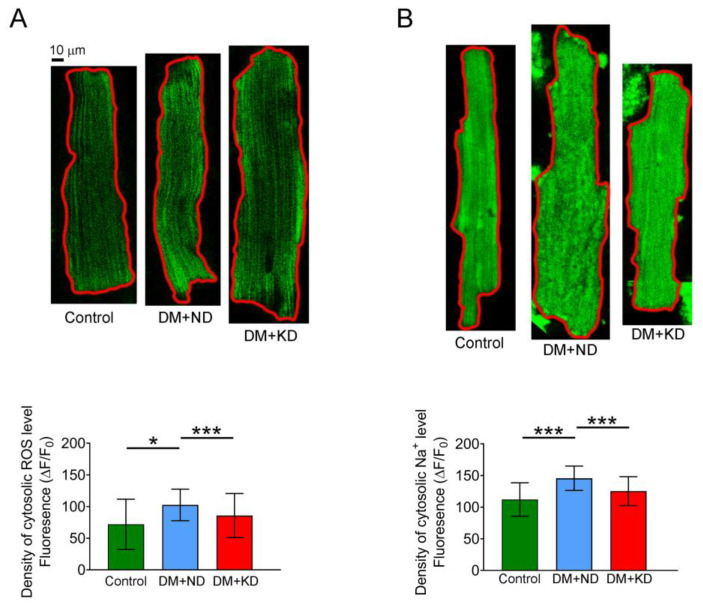
Oxidative stress and cytosolic sodium (Na^+^) levels in the ventricular myocytes of the control rats (control), diabetes mellitus (DM) rats on a normal diet (DM + ND), and DM rats on the ketogenic diet (DM + KD). (**A**) Average data of cytosolic ROS levels and examples of cytosolic ROS in the ventricular myocytes of the control (n = 31), DM + ND (n = 54), and DM + KD (n = 24) rats. Scale bar, 10 μm. (**B**) Average data of cytosolic Na^+^ levels and an example in the ventricular myocytes of the control (n = 22), DM + ND (n = 42), and DM + KD (n = 42) rats. Scale bar, 10 μm Abbreviations: ROS: reactive oxygen species, n: number of cardiomyocytes isolated from the rats. Statistical significance was assessed using a one-way analysis of variance (ANOVA) with Tukey’s honest significant difference (HSD) test. * *p* < 0.05; *** *p* < 0.005.

As shown in Figure 6, cytosolic reactive oxygen species (ROS) levels were onefold higher in the ventricular myocytes of DM rats on ND (102.6 ± 39.6 ΔF/F_0_, n = 54) than in the control rats (72.0 ± 39.6 ΔF/F_0_, n = 32; Tukey’s HSD test: *p* < 0.005) and DM rats on the KD (85.8 ± 34.8 ΔF/F_0_, n = 24; Tukey’s HSD test: *p* < 0.05). The cytosolic ROS levels of the control and DM rats on KD ventricular myocytes were similar (Figure 6). Similarly, intracellular Na^+^ ([Na^+^]_i_) levels in ventricular myocytes were 1.5-fold higher in DM rats on an ND (145.7 ± 19.3 ΔF/F_0_, n = 42) than in the control rats (112.1 ± 26.4 ΔF/F_0_, n = 22) and DM rats on the KD (125.4 ± 22.8 ΔF/F_0_, n = 42; Tukey’s HSD test, both *p* < 0.005; Figure 6). Control rats demonstrated 1.2-fold higher [Na^+^]_i_ levels in ventricular myocytes than DM rats on the KD.

### 2.6. The Effects of Ca^2+^ Regulatory Proteins in DM Hearts with or without a KD

**Figure 7:**
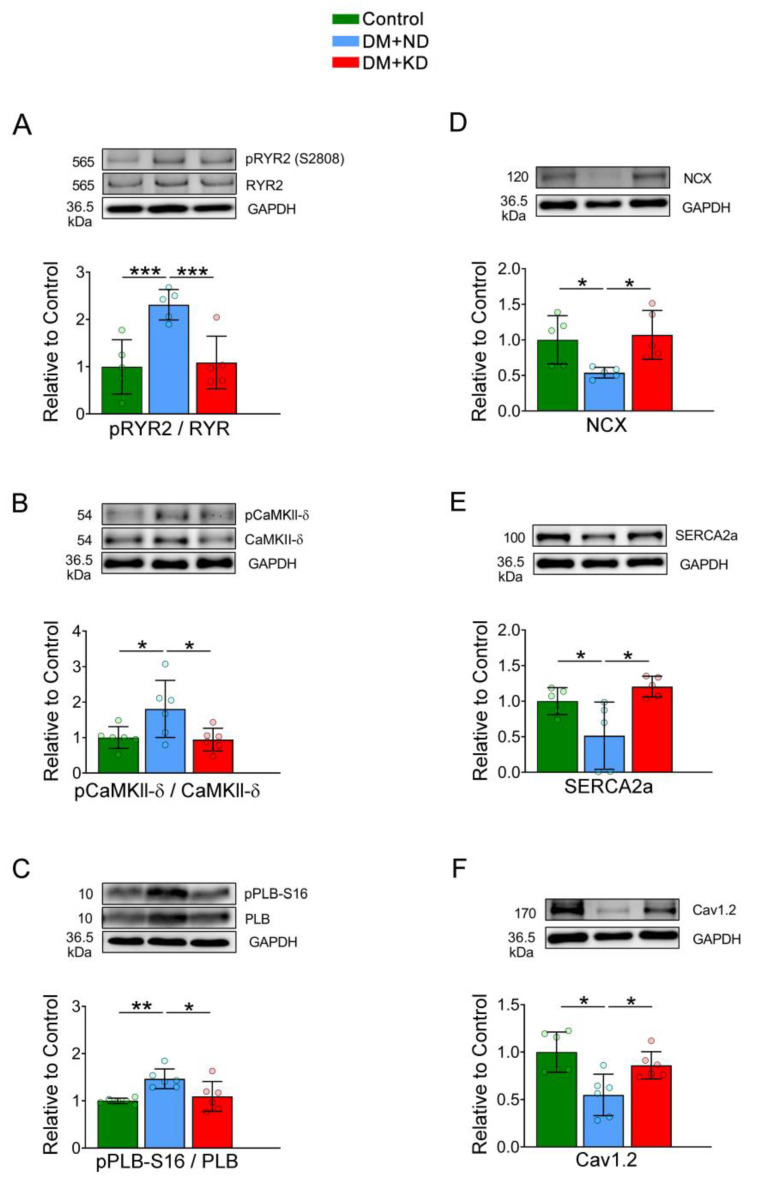
Calcium regulating proteins in the ventricular myocytes of the control rats (control), diabetes mellitus (DM) rats on a normal diet (DM + ND), and DM rats on a ketogenic diet (DM + KD). Average data and representative immunoblots of (**A**) pRyR2 at serine 2808/RyR2 ratio (N = 6 per group), (**B**) pCaMKIIδ/CaMKII-δ (N = 6 per group), (**C**) pPLB-S16/PLB (N = 6 per group), (**D**) NCX (N = 5 per group), (**E**) SERCA2a (N = 6 per group), and (**F**) Cav1.2 (N = 6 per group) in the ventricular myocytes of the control, DM + ND, and DM + KD rats. Densitometry was normalized to GAPDH as the internal control. Abbreviations: RyR2: ryanodine receptor 2, pRyR2: phosphorylated RyR2, NCX: sodium/calcium exchanger, SERCA2a: sarcoplasmic reticulum calcium ATPase, Cav1.2: voltage-dependent L-type alpha 1C subunit, CaMKII-δ: calcium/calmodulin-dependent protein kinase II, pCaMKII-δ: phosphorylated CaMKII-δ, PLB: phospholamban, pPLB: phosphorylated PLB. N: number of rats. Statistical significance was tested using a one-way analysis of variance (ANOVA) with Tukey’s honest significant difference (HSD) test. * *p* < 0.05, ** *p* < 0.01, *** *p* < 0.005.

The expression of Ca^2+^ regulatory proteins in the ventricles of the control rats, DM rats on an ND, and DM rats on the KD were analyzed using Western blotting (Figure 7). Compared with DM rats on an ND, those on the KD exhibited increased expressions of SR Ca^2+^-ATPase (SERCA2a), NCX, and voltage-dependent L-type alpha 1C subunit (Cav1.2) (Figure 7). Furthermore, DM rats on an ND demonstrated elevated ratios of phosphorylated ryanodine receptor 2 (pRyR2) at serine 2808/RyR2, phosphorylated phospholamban (pPLB)-Ser 16/PLB, and phosphorylated Ca^2+^/calmodulin-dependent protein kinase II (pCaMKII-δ)/CaMKII-δ compared to both the control and DM rats on the KD (Figure 7).

## 3. Discussion

A KD may have cardiovascular benefits in DM hearts [4,16,17]; however, the results have been controversial [18,19]. In DM hearts, changes in the excitation–contraction coupling have been noted, which include defects in [Ca^2+^]_i_ signaling [20], SERCA2a [21], RyR2 [22], and NCX [23]. However, whether a KD can ameliorate the electromechanical dysfunction in diabetic cardiomyopathy remains unknown. We discovered in this study that KD administration reversed the electromechanical dysfunction of the hearts of DM rats. More specifically, a KD attenuated DM’s effect on ventricular APs, [Ca^2+^]_i_ transients, and I_Na-late_, which may be electromechanical mechanisms underlying the antidiabetic cardiomyopathy effects of a KD.

DM patients generally demonstrate a high incidence of prolonged QT and QTc interval because of an increment in the APD and repolarization, making them liable to have an increased incidence of cardiac arrhythmia [24,25]. Several studies have also demonstrated that atrioventricular conduction disturbances and sinus node dysfunction occur more frequently in DM patients [26,27]. Like our earlier studies [12,28], the current DM rats on an ND showed a higher heart-to-body weight ratio than the control and DM rats on a KD. This could have been due to severe hyperglycemia in the DM rats on an ND, reducing their body weight; thus, the ratio to heart weight was more prominent compared to the other two groups. Additionally, DM rats on an ND had a prolonged ventricular APD and a longer QT and QTc interval. The prolonged APD and QT interval aggravates the reduction in the stroke volume and diastolic filling at a higher heart rate [29]. Ventricular arrhythmia may increase because early afterdepolarization activity will be triggered [28]. The 2020 Dietary Guidelines Advisory Committee concluded that dietary patterns that included low-fat dairy are correlated with a lesser risk of all-cause mortality, overweight, obesity, and cardiovascular disease. However, it is essential to note that the advantages of low-fat and fat-free dairy products compared with full-fat dairy products are not without arguments and continue to be controversial [30]. In our present study, a KD attenuated the prolonged APD and QTc intervals in DM hearts, suggesting that a KD might have a cardioprotective potential against diabetic cardiomyopathy. However, clinical data supporting the beneficial effects of a KD on heart health remains limited [31,32].

Ca^2+^ is responsible for the connection between mechanical contraction and electrical activation and is crucial in excitation–contraction coupling. Ca^2+^ from its sarcoplasmic reticulum (SR) stores through RyR2 triggers the release of ionic Ca^2+^. The ionic Ca^2+^ further facilitates Ca^2+^ binding to the myofilaments, enhances [Ca^2+^]_i_ content, and activates myocardium contractions [33]. Similar to our previous studies [12,28], we noted depletion of SR Ca^2+^ content associated with prolonged [Ca^2+^]_i_ decay and relatively diminished [Ca^2+^]_i_ transients in DM hearts. According to a previous study, a reduction in SR Ca^2+^ uptake is one of the primary mechanisms causing a diminution in the myocardial contractility of diabetic cardiomyopathy [34]. In our DM rats’ ventricular myocytes, the dysfunction in SERCa2α may be ascribed to a reduced protein expression of SERCA2a, leading to transient decay rates and decreased [Ca^2+^]_i_ transient amplitudes [21,35]. In our DM cardiomyocytes, reduction in [Ca^2+^]_i_ transients may have originated from the reduced SR Ca^2+^ content, impairing excitation–contraction coupling efficiency. The impaired reuptake of Ca^2+^ during the diastolic phase may have caused SERCA function impairment, causing a decline in SR Ca^2+^ stores, which might have worsened the condition of our DM rat hearts due to lowered SERCA2a levels. The decrease in SERCA2a levels might have reduced SR Ca^2+^ stores in our DM rat hearts. The prolonged [Ca^2+^]_i_ transient decay in our DM rat hearts might also have been due to SR Ca^2+^ depletion. The rise in the transient decay of [Ca^2+^]_i_ might be attributed to the dysfunction of the SERCA pump, generating a defect in cardiomyocyte relaxation and a slower removal rate of cytoplasmic Ca^2+^ [23,36].

An increase in [Ca^2+^]_i_ transient decay may also result from prolonging APD in DM ventricular myocytes. We also discovered that a KD reversed the decreased [Ca^2+^]_i_ transients and delayed [Ca^2+^]_i_ decay in DM hearts, which diminished the Ca^2+^ storage in DM hearts. An augmented SERCA2a function in the myocardium could have caused these effects. Furthermore, as assessed through echocardiography, the modulation of Ca^2+^ by the KD in DM hearts likely significantly mitigates diabetic cardiomyopathy. In our study, a KD alleviated Ca^2+^ dysregulation caused by DM, as evidenced by reduced phosphorylation of both RyR2 at serine 2808 and CAMKII-δ and increased SERCA2a expression. These findings suggest that a KD improves Ca^2+^ homeostasis, increasing EF in DM hearts.

I_Ca-L_ plays a vital role in inducting the contractile cycle of cardiomyocytes. Inhibition of I_Ca-L_ reduces the entry of Ca^2+^, resulting in a diminution in contractile force and Ca^2+^ transients [37]. A decreased I_Ca-L_ in the ventricular myocytes of our DM rats significantly lowered peak systolic [Ca^2+^]_i,_ similar to our previous results [35,38]. Cav1.2 is the principal pathway to enter Ca^2+^ into the myocardial cells [39]. In our DM cardiomyocytes, Cav1.2 is also altered based on the decreased levels of Cav1.2. Decreased I_Ca-L_ density and Cav1.2 expression were restored to control levels in DM rats after KD administration, suggesting that a KD may have a cardioprotective role against diabetic cardiomyopathy.

Comparable with the outcomes from other investigations [40,41,42], our DM rat cardiomyocytes, through voltage-dependent I_Ca-L,_ demonstrated diminished Ca^2+^ entry. In our DM rat hearts, the adverse effects on ventricular contractility were caused mainly by the decreased availability of Ca^2+^ through the I_Ca-L_ channel. As indicated in electrocardiograms, this may have led to prolonged QT and QTc intervals. However, the decreased I_Ca-L_ in our DM rat hearts was ameliorated in DM rats on the KD, contributing to the increase in SR Ca^2+^ content and [Ca^2+^]_i_ transients and suggesting the cardioprotective effect of a KD in diabetic cardiomyopathy. Similarly, we found that NCX function is reduced in DM cardiomyocytes. Inhibition of NCX in its reverse mode, which mediates Ca^2+^ influx, might have restricted the cellular Ca^2+^ content, subsequently contributing to a reduction in [Ca^2+^]_i_ and leading to electrical dysfunction. The KD effectively improved the reduced NCX current in the ventricular myocytes of DM hearts.

Moreover, elevation in Na^+^/H^+^ exchanger current and I_Na-late_ in our DM rats cardiomyocytes may have contributed to the rise in [Na^+^]_i_, causing Ca^2+^ overload and increasing the risks of oxidative stress and arrhythmia [43]. Our results showed that a KD reduced intracellular Na^+^ concentrations and turned back I_Na-late_ and Na^+^/H^+^ exchange currents. These findings suggest that a KD has cardiovascular benefits in DM hearts.

In the heart’s excitation–contraction coupling, RyR2, a macromolecular homotetrameric protein complex, controls the release of Ca^2+^ from the SR [39]. CaMKII, one of the accessory proteins involved in RyR2 regulation, interacts with RyR2 and stabilizes the channel, reducing spontaneous Ca^2+^ release and SR Ca^2+^ leak [44]. Similar to others, we also noted a significant increment in the phosphorylated RyR2 at serine 2808/total RyR in our DM rats’ ventricular myocytes [45,46]. The KD may have increased [Ca^2+^]_i_ transient amplitude, improved myocardial contractility, and reversed RyR2 hyperphosphorylation in our DM hearts. As observed in our DM rats’ ventricular myocytes, a decrease in RyR2 function prolongs the time to peak Ca^2+^ transients and slows down Ca^2+^ release [36]. Because of enhanced RyR2 channel activity in DM hearts, the rise in the SR Ca^2+^ leak facilitates their arrhythmogenic potential [47]. Increased Ca^2+^ leak in our investigation might have been associated with increased phosphorylated RyR2 at serine 2808/total RyR2 ratio, causing the reduction in SR Ca^2+^ content and leading to diabetic cardiomyopathy. Chronic [Ca^2+^]_i_ leak from cardiomyocytes causes an increase in endoplasmic reticulum stress and mitochondrial dysfunction. Mitochondrial dysfunction also increases ROS production and provokes redox modifications of RyR2, thereby exacerbating the leakage of Ca^2+^ [48]. Consequently, [Ca^2+^]_i_ leak inhibition is a critical mechanism of action of the KD in DM rats. Thus, a KD may help reduce excessive Ca^2+^ leakage in the SR, resulting in a Ca^2+^ shortage in diabetic cardiomyopathy.

Heart disorders, including cardiac hypertrophy, arrhythmias, and heart failure, are linked to CaMKII overexpression [49]. CaMKII is an essential mediator for excitation–contraction coupling in the heart because it regulates Ca^2+^ regulatory proteins [50]. CaMKII activation can cause alterations in [Ca^2+^]_i_ signaling, causing impairments in transcriptional regulation, cell mechanics, energetics, and excitation–contraction coupling [49,51]. CaMKII-δ alters the regulation of Ca^2+^ by increasing phosphorylating PLB and RyR2, affecting the SR Ca^2+^ content to fluctuate and causing a leak of diastolic Ca^2+^ [52], leading to diastolic dysfunction and an increase in arrhythmogenesis [53]. CaMKII-δ inhibition, evidenced by the decrease in phosphorylated CaMKII-δ/total CaMKII-δ ratio after KD administration, reduces diastolic SR Ca^2+^ leak in DM rats on the KD, resulting in a diminution in the released of spontaneous Ca^2+^ and an improvement in the SR’s capacity to store Ca^2+^. This suggested the protective role of a KD against arrhythmogenicity. Moreover, a KD reduces both [Na^+^]_i_ level and CaMKII activity, contributing to reductions in cardiac remodeling related to diabetic cardiomyopathy.

We also investigated ROS generation in DM hearts and noted that a KD reduces cytosolic ROS production. ROS can cause local endoplasmic reticulum Ca^2+^ release events, enhancing the frequency of sparks and Ca^2+^ leaks in the cardiomyocytes. The reduced ROS generation following KD ingestion may have helped to decrease the Ca^2+^ leak in DM cardiomyopathy. Additional assessment of the mechanical functions to comprehend the cardioprotective impact of a KD is necessary for DM cardiomyopathy. The decrement of Ca^2+^ leak, ROS, I_Na-late_, and Na^+^/H^+^ exchanger current in the DM cardiomyocytes on the KD suggests that a KD may have antiarrhythmic potential. Therefore, further investigation to establish the antiarrhythmic effect of a KD is necessary.

Hyperglycemia is linked with a high risk of arrhythmia and myocardial dysfunction. Several studies have confirmed that a KD has beneficial effects on enhancing body weight loss [54], glycemic control [55], and other cardiovascular risk factors such as blood pressure [56] and lipid profiles [57,58]. However, some precautions need to be addressed. Severe hypoglycemia in patients with DM taking insulin or oral hypoglycemic agents if medications are not appropriately adjusted before a KD [59]. A KD should be avoided in patients taking SLGT2i to avoid euglycemic diabetic ketoacidosis [60,61]. In addition, a KD is contraindicated in patients with fat metabolism disorders, liver failure, pancreatitis, and deficiencies in carnitine, carnitine palmitoyltransferase, carnitine translocase, porphyrias, or pyruvate kinase [57,62]. Our study showed several potential factors related to the regulation of Ca^2+^, which are dysregulated in diabetic cardiomyopathy, including altered expressions of RyR2, phospholamban, SERCA2a, NCX, and CaMKII-δ, and levels of I_Ca-L_ channel activity. High cytosolic concentrations of Ca^2+^ have been demonstrated to induce early cell death. Additionally, changes in mitochondrial Ca^2+^ handling may lead to mitochondrial respiratory dysfunction and subsequent cell death [63]. Metabolic stress-induced mitochondrial dysfunction also contributes to Ca^2+^ overload, triggering autophagy and cardiac necrosis in cardiomyocytes [64]. Impaired Ca^2+^ handling in DM cardiomyocytes is likely a critical factor in the progression of cardiac diastolic dysfunction, a hallmark of early diabetic cardiomyopathy. The KD in our study exhibited a dual beneficial effect in the context of diabetic cardiomyopathy. The KD improved Ca^2+^ homeostasis by reducing PLB (which inhibits SERCA2a activity) and increasing SERCA2a. This enhancement in Ca^2+^ uptake into the SR in DM cardiomyocytes suggests a potential reduction in cell death of myocytes. However, direct measurement of cell death was not part of our study. Additionally, regulating other Ca^2+^ handling proteins, including RyR2, NCX, and CaMKII-δ, under a KD suggests potential antiarrhythmic properties. These alterations collectively may improve cardiac cell function in DM conditions. The novelty of our study lies in emphasizing the roles of a KD in protecting against cardiac electrical and structural remodeling and in potentially mitigating diabetic cardiomyopathy through its dual action on Ca^2+^ handling proteins, which might indirectly attenuate cell death. This discovery offers mechanistic insights into diabetic cardiomyopathy’s etiology and might result in more effective tailored treatments for DM patients.

**Figure 8:**
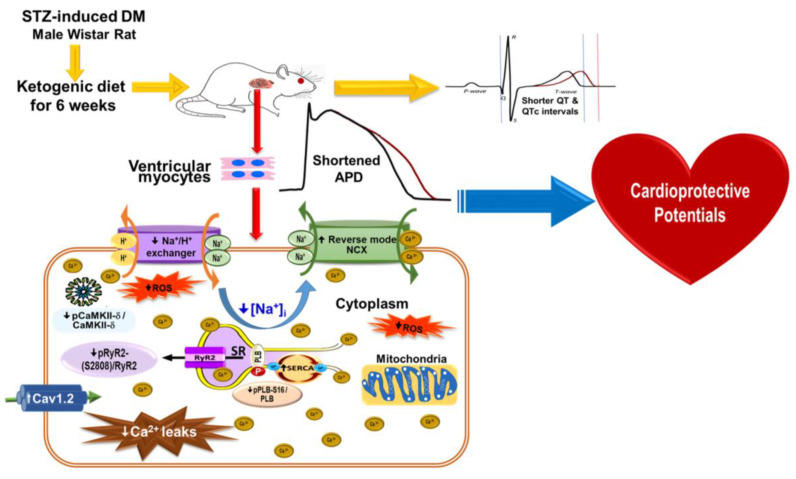
Schematic diagram of the proposed mechanism of action underlying the effects of a KD in DM hearts. A KD may reverse DM-induced Ca^2+^/Na^+^ dysregulation by reducing ROS and ionic channel modification in the cardiomyocytes, leading to the correction of prolonged QTc intervals and improvement in diabetic cardiomyopathy. Abbreviations: APD: action potential duration, DM: diabetes mellitus, Ca^2+^: calcium, Cav1.2: Ca^2+^ channel, voltage-dependent, L-type alpha 1C subunit, H^+^: hydrogen, [Na^+^]_i_: intracellular sodium, I_Na-Late_: late sodium current, Na^+^: sodium, NCX: Na^+^/Ca^2+^ exchanger, QTc: corrected QT interval, ROS: reactive oxygen species, RyR2: ryanodine receptor 2, pRyR2: phosphorylated RyR2, SR: sarcoplasmic reticulum, SERCA: sarcoplasmic reticulum ATPase, STZ: streptozotocin.

Figure 8 details the possible mechanisms underlying the effects of a KD in rats with diabetic cardiomyopathy: a KD may restore Ca^2+^/Na^+^ regulation by modifying ionic channels in a DM heart, resulting in the alleviation of ventricular hypertrophy, reversion of QT interval prolongation, and amelioration of diabetic cardiomyopathy.

### Study Limitation

In this study, the KD was implemented at the beginning of DM creation. However, the optimal timing for initiating a KD as a therapeutic approach to improve diabetic cardiomyopathy has not been established, and more clinical studies are needed to provide robust evidence. Furthermore, our assessment of myocardial function was restricted to basic echocardiographic measurements. A more precise analysis of LV dysfunction, with or without systolic dysfunction, could have been achieved if tissue Doppler imaging had been performed. Moreover, inflammatory pathways and mediators activate the structural and functional changes and Ca^2+^ homeostasis of diabetic cardiomyopathy [65,66]. A KD has been shown to modulate immunity in cardiovascular diseases [67]. However, whether a KD may change DM cardiomyopathy via its modulatory effects on immune response remains unclear.

## 4. Material and Methods

The Supplementary Materials available online reports more thorough procedures.

### 4.1. Animal Experiments

**Figure 9:**
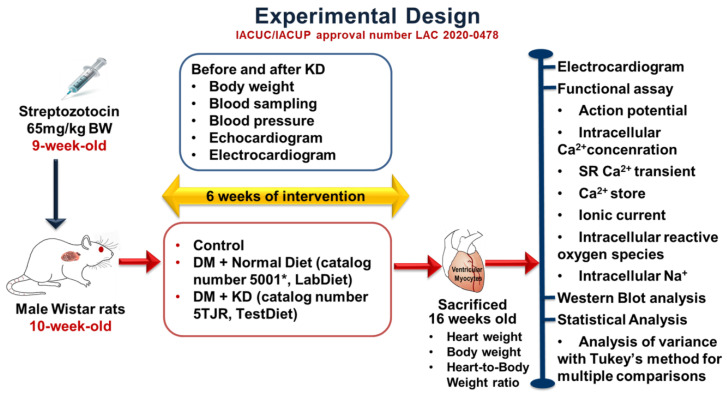
Schematic of our experimental design. The IACUC approval number is LAC 2020-0478. Abbreviations: KD: ketogenic diet, DM: diabetes mellitus, Ca^2+^: calcium, SR: sarcoplasmic reticulum, Na^+^: sodium.

As shown in Figure 9, the Institutional Animal Care and Use Committee Panel of Taipei Medical University reviewed and approved our animal experimentation protocols with the IACUC/IACUP approval number LAC 2020-0478. The Guide for the Care and Use of Laboratory Animals (eighth edition), published by the US National Academies Press (NBK54050) in 2011, was also followed. All animal works were conducted in the TMU Laboratory Animal Center located at Taipei Medical University. Wistar male rats at 8 weeks of age were maintained in a controlled setting with a temperature of 21.2 °C, a 12 h light–dark cycle with unlimited commercial rat food and water. At 9 weeks old, a part of the male Wistar rats had DM induced after 10 h of overnight fasting, using a single intraperitoneal injection of streptozotocin (STZ, 65 mg/kg; Sigma-Aldrich, St. Louis, MO, USA) [28,68]. A fasting blood glucose of ≥15 mmol/L was used to diagnose DM, similar to our previous investigation [28,68], using a glucometer (Bayer Contour PLUS Glucometer, Ascensia Diabetes Care, NJ, USA). The male Wistar rats were randomly grouped into the control group (ND), the DM with ND group, and the DM with KD group at 10 weeks age for six weeks. The energy content of the ND (catalog number 5001*, Rodents, LabDiet, St. Louis, MO, USA) was approximately 3.35 kcal/g, 28.9% protein, 13.6% fat, and 57.5% carbohydrates. While the energy content from the KD (catalog number 5TJR, Rodents, Test Diet, St. Louis, MO, USA) was around 5.29 kcal/g, 65.3% fat, 2% carbohydrates, and 32.6% protein (Supplementary Table S2). Body weights and fasting blood glucose levels were recorded weekly. The average diet consumed per rat was 31.4 g from the ND and 20.0 g from the KD group. The average daily energy intake per rat was around 105 kcal between the ND and KD.

Electrocardiography was conducted initially at the age of 10 weeks and subsequently at 16 weeks to assess cardiac function before and after the dietary intervention, respectively, prior to euthanasia. Rats were anesthetized by inhalation using 2.0–2.5% isoflurane (5% in oxygen; Panion & BF biotech, Taoyuan, Taiwan). Their hemodynamics were stabilized after 30 min under continuous electrocardiography monitoring. The electrocardiogram tracings were collected using Bio-amplifier’s standard lead II limb with an ML 845 Powerlab polygraph recorder AD Instruments (Castle Hill, Australia) [69]. The readings of the control and DM rats on the KD and he ND were recorded continually.

The male Wistar rats were initially sedated with zoletil (10 mg/kg; Virbac, Carros, France) and xylazine (5 mg/kg; Bayer, Leverkusen, Germany) intramuscularly. Then, they were terminated with an overdose of isoflurane inhalation with a precision vaporizer (5% in oxygen; Panion & BF biotech, Taoyuan, Taiwan) [12,70]. The dosage of anesthesia was confirmed to be adequate based on the absence of chest movement, palpable heartbeat, corneal reflexes, and no response to toe pinch. The hearts were removed through midline thoracotomy for further electrophysiological, confocal microscopy, and Western blot experiments.

### 4.2. Cardiomyocyte Isolation

Single LV myocytes were isolated from adult male Wistar rats using a Langendorff perfusion setup. The heart and the lungs were removed from the anesthetized rats after mid-line thoracotomy. In a retrograde manner, the heart was perfused through a polyethylene tubing cannulated along the aorta. Perfusion was carried out with oxygenated standard Tyrode’s solution at 37 °C, comprising KCl 5.4, NaCl 137, MgCl_2_ 0.5, CaCl_2_ 1.8, glucose 11 mM, and HEPES 10; pH was adjusted to 7.4 using 1 N NaOH. The perfusate was then switched to oxygenated Ca^2+^-free Tyrode’s solution consisting of 0.25 units/mL protease (Sigma, Type XIV) and 300 units/mL collagenase (Sigma Type I) for 6–8 min. Subsequently, the LVs were separated from the lungs and atrium by gently swaying in 5–10 mL of Ca^2+^-free oxygenated Tyrode’s solution until single LVs were attained. The mixture fluid was then carefully replaced with normal oxygenated Tyrode’s solution. The isolated cells showed a classical cylindrical shape with well-discernible cross striations. The size of these cells ranged from 10 to 25 µm in width and around 80 to 130 µm in length. Only isolated cells exhibiting cross striations were used for the investigation.

### 4.3. Measurement of Intracellular Ca^2+^ ([Ca^2+^]_i_), SR Ca^2+^ Transient, and Ca^2+^ Store

The [Ca^2+^]_i_ was evaluated based on the fluorometric ratio (fluo-3 fluorescence). Briefly, the freshly isolated single LV myocytes were plated on glass coverslips (diameter = 25 mm), which were pre-coated with poly-L-lysine solution (0.01% *w*/*v*) for one hour. No Ca^2+^ agonist was used. Cells (around 50–60% confluent) were loaded with a fluorescent Ca^2+^ indicator fluo-3/AM (10 μM) for 30 min in standard Tyrode’s solution at room temperature. Excess extracellular dye was removed by changing the bath solution and allowing the intracellular hydrolysis of fluo-3/AM after 30 min. At >515 nm, emission was confirmed with an argon-ion laser 3 fluorescence stimulated at 488 nm. The cells were repeatedly scanned at 2 ms intervals using a line scan (8-bit) imaging. Fluorescent cells were monitored using Zeiss LSM 510 (Carl Zeiss, Jena, Germany) laser scanning confocal microscope and an Axiovert 100 inverted microscope (Carl Zeiss, Jena, Germany). The transient [Ca^2+^]_i_ changes were calculated by measuring the fluorescent signals and baseline values (F/F_0_). The fluorescence of fluorescent signals (F) was compared to that of the baseline fluorescence (F_0_), variations in dye concentrations were corrected, and fluorescence intensity variations generated by various dye injection volumes were excluded [71]. [Ca^2+^]_i_ transients, diastolic [Ca^2+^]_i_, peak systolic [Ca^2+^]_i_, and decaying fraction [Ca^2+^]_i_ transients were monitored using 10 ms twice-threshold strength square-wave pulses after 1-Hz field stimulation. Moreover, the monoexponential least-squares fit was used to calculate [Ca^2+^]_i_. After a steady-state [Ca^2+^]_i_ transient was achieved with multiple pulses, the superfusate containing tetracaine (1 mmol/L) for 20 s was changed to 0 Na^+^/0 Ca^2+^ solutions (1 Hz for 15 s). The 1 mmol/L tetracaine-reduced [Ca^2+^]_i_ was used to quantify the SR Ca^2+^ leak as previously described [72].

Fast determination of SR Ca^2+^ reserves were measured using a 30 s pulse stimulation train at 1 Hz followed by the addition of 20 mmol/L of caffeine. The SR Ca^2+^ reserve was calculated from the peak amplitude of the caffeine-induced Ca^2+^ transient. An amount of 20 mmol/L of caffeine voltage-clamped at −40 mV was quickly injected into cells. As previously mentioned, [73,74,75], the SR Ca^2+^ level was calculated using the integral of the inward Na^+^/Ca^2+^ exchanger (NCX) current as follows:

SR Ca^2+^ content (mol/L/L cytosol) = [(1 + 0.12 Ccaff/F × 1000)]/(Cm × 8.31 × 8.44). In addition to the cell surface-to-volume ratio being 8.44 pF/pL, Ccaff is the integral of the inward NCX current caused by caffeine, F is Faraday’s number, and Cm is the capacitance of the membrane.

### 4.4. AP and Ionic Currents

An Axopatch 1D amplifier (Axon Instruments, Foster City, CA, USA) at 35 ± 1 °C was used on the newly isolated rat ventricular myocytes to perform whole-cell patch-clamp. In the voltage-clamp mode, ionic currents were reported, and action potentials (APs) directed at 1 Hz in the current-clamp mode were identified. I_Ca-L_ was measured using micropipettes filled with a solution composed of 130 mM CsCl, 1 mM MgCl_2_, 5 mM MgATP, 10 mM HEPES, 5 mM Na^2+^ phosphocreatine, 10 mM EGTA, and 0.1 mM NaGTP. The NCX current was measured with 110 mM CsCl, 20 mM NaCl, 1.75 mM CaCl_2_, 20 mM tetraethylammonium, 0.4 mM MgCl_2_, 10 mM HEPES, 5 mM 1,2-bis (2-amino phenoxy) glucose, 75 mM 1,2-bis(2-amino phenoxy) ethane-N,N,N′,N′-tetraacetic acid, and 5 mM MgATP titrated to a pH of 7.25. The late sodium current (I_Na-Late_) was measured using 130 mM CsCl, 10 mM NaCl, 5 mM HEPES, 5 mM EGTA, 5 mM MgATP, and 5 mM glucose. The action potential (AP) was measured using 110 mM K aspartate, 20 mM KCl, 5 mM MgATP, 1 mM MgCl_2_, 0.5 mM EGTA, 10 mM HEPES, 5 mM Na^2+^ phosphocreatine, and 0.1 mM LiGTP titrated with KOH to a pH of 7.2. A digital-to-analog converter (12-bit) was used to create voltage command pulses on a pCLAMP (Axon Instruments). A low-pass filtration was applied to the recordings during half the sampling frequency. The AP amplitude (APA) was assessed as the difference between the peak AP depolarization and resting membrane potential (RMP). The repolarization of APD_90_, APD_50_, and APD_20_ were calculated.

I_Ca-L_ was measured during depolarization from a holding potential of −50 mV at 0.1 Hz as an inward current to evaluate the possibilities of −40 to +60 mV in 10 mV increments for 300 ms. CsCl and tetraethylammonium chloride were used with the external solution instead of NaCl and KCl. We used pulses between −100 and +100 mV given over 300 ms at depolarizing of 0.1 Hz to produce the current of NCX. Nickel-sensitive currents with a 10 mM nickel Cl were used to quantify NCX current amplitudes. The external solution contained 5 mM HEPES, MgCl_2_, 10 mM glucose, and 140 mM NaCl. Additionally, the external solution had dihydropyridine antagonist, 10 μM nitrendipine, 10 μM strophantidin to inhibit Na^+^/K^+^ pump, and 100 μM niflumic acid to impede Ca^2+^-activated Cl currents, and the pH was adjusted to 7.4.

Using a step/ramp procedure (where −100 mV was stepped to +20 mV at ambient temperature for 100 ms and then ramped back to −100 mV over 100 ms), I_Na-Late_ was documented with an external solution comprising 130 mM NaCl, 5 mM CsCl, 1 mM CaCl_2_, 10 mM HEPES, 1 mM MgCl_2_, and 10 mM glucose. I_Na-Late_ current was determined when the current was produced and when the voltage was ramped back to −100 mV and was sensitive to 30 μM tetrodotoxin as described previously [76].

An internal solution comprising 130 mM K-aspartate, 20 mM KCl, 1 mM MgCl_2_(6 H_2_O), 0.005 mM EGTA, and 10 mM HEPES/KOH (pH 7.3) was used to evaluate the Na^+^/H^+^ exchanger current in a whole-cell model. The external solution contained 3.6 mM CaCl_2_, 150 mM NaCl, 1.2 mM MgCl_2_(6 H_2_O), 5.4 mM KCl, 20 mM glucose, and 5 mM HEPES/NaOH (pH 7.4). At 3 Hz, rat ventricular myocytes were isolated, and 40 ms depolarizing pulses from −45 to 0 mV were used [77].

### 4.5. Intracellular Na^+^ and Reactive Oxygen Species (ROS) Measurements

The ROS production in the mitochondria and cytosol of the isolated rat LV myocytes was evaluated using CellROX green (Life Technologies). Moreover, cytosolic Na^+^ concentration in the ventricular myocytes was measured using Asante NaTRIUM Green-2 AM (Teflabs). Like our previous investigation [78], an experiment was performed using an Axiovert 100 inverted microscope with a 63×/1.25 numerical aperture oil immersion objective and a Zeiss LSM 510 laser scanning confocal microscope (Carl Zeiss). The isolated single LV myocytes were placed in a standard Tyrode solution containing 5.4 mM KCl, 137 mM NaCl, 0.5 mM MgCl_2_, 10 mM HEPES, 1.8 mM CaCl_2_, and 5.6 glucose at pH 7.3. Then, these isolated single LV myocytes were incubated for 25 min at 23 °C with an appropriate fluorescent dye, either 10 μM CellROX Green, or 5 μM Asante NaTRIUM Green-2 AM (Teflabs).

Similar to other investigations [78], CellROX Green were stimulated at 488 nm, and fluorescence signals were acquired at wavelengths >505 nm using the XY mode of the confocal system. Asante NaTRIUM Green-2 AM was excited at 543 nm, and fluorescence was acquired at wavelengths >560 nm in the XY scan mode. Moreover, the myocytes were timed at 1 Hz during the experiment. Using Sigma Plot (version 12) and Image-Pro Plus (version 6.0), fluorescence images were examined as described previously [12].

### 4.6. Western Blot

The LV tissues were first homogenized using a homogenizer and extracted with M-PER™ Mammalian Protein Extraction Reagent (Thermo Scientific, Waltham, MA, USA), supplemented with Protease and Phosphatase Inhibitor (Thermo Scientific). Subsequently, the proteins were centrifuged at 13,500 rpm for 30 min. Following protein extraction, their concentrations were determined using the QubitTM Protein Assay Kit (Invitrogen). These proteins were then boiled for 5 min, and each lane was loaded with 30 µg of protein. These proteins were separated using standard sodium dodecyl-sulfate polyacrylamide gel electrophoresis (SDS-PAGE), utilizing 8% SDS-PAGE or 15% SDS-PAGE, depending on the size of the target protein. The running buffer (25 mM Tris-base, 192 mM Glycine, 0.1% SDS) was placed at 95 v for about 90 min.

After electrophoresis, the separated proteins were transferred to a polyvinylidene fluoride (PVDF; Immobilon Transfer Membranes, 0.45 µm, EMD Millipore) membrane in a transfer buffer (25 mM Tris-base, 192 mM Glycine, 20% methanol) at 70 v for 120 min [15]. Blocking was carried out using 5% skim milk in phosphate-buffered saline solution with Tween (PBST; PBS containing 0.1% Tween 20) at room temperature for 60 min. After that, washing with PBST 3 times (5 min per wash) was carried out. After blocking, the primary antibodies were bound to the target protein by diluting in 5% PBST and overnight at 4 °C. Thenceforth, washing with PBST 3 times (5 min per wash) was carried out. The obtained blots were incubated with antibodies against RyR2 (1:5000; MA3-916; Affinity Bioreagents, Golden, CO, USA), phosphorylated RyR2 (pRyR2 (S2808); 1:3000; A010-30AP; Badrilla, Leeds, UK), Ca^2+^ channel, Cav1.2 (1:1000; Acc-003; Alomone Labs, Jerusalem, Israel), NCX (1:3000; Swant, Bellizona, Switzerland), SERCA2a (1:1000; Sc-376235; Santa Cruz Biotechnology, CA, USA), CaMKII-δ (1:2000; GTX111401; Gene Tex, CA, USA), pCaMKII-δ (1:2000; ab32678; Abcam, Cambridge, UK), PLB (1:5000; MA3-922; Thermo Fisher Scientific, MA, USA), and phosphorylated PLB (pPLB)-S16; (1:5000; A010-12; Badrilla, Leeds, UK). The blots were then exposed to the specific enzyme-conjugated secondary antibody in diluted 5% skim milk in PBST at room temperature for 60 min. Hereafter, washing with PBST 3 times (5 min per wash) was performed. Bound antibodies were detected using an electrochemiluminescence (ECL) system. Band intensities were measured using Alpha Innotech by AlphaEaseFC (San Leandro, CA, USA) Gbox Chemi-XL (Syngene; Worcester, MA). Glyceraldehyde 3-phosphate dehydrogenase (GAPDH; M171-7; Sigma-Aldrich) was used to standardize and validate equal protein loaded from the targeted bands as described previously [79,80,81].

### 4.7. *Statistical Analysis*

All quantitative data are expressed as the mean ± standard deviation (SD). A one-way analysis of variance (ANOVA) with Tukey’s HSD test was conducted (GraphPad Prism version 8.0, San Diego, CA, USA) to identify the significant between-group differences. Statistical significance was defined as a *p*-value of <0.05.

## 5. Conclusions

In conclusion, as summarized in Figure 8, DM-induced Ca^2+^ or Na^+^ dysregulation in the cardiomyocytes was restored in the hearts of KD-treated rats by reducing ionic channel modification and ROS, resulting in decreased ventricular hypertrophy and prolonged QT interval correction in diabetic cardiomyopathy.

## Data Availability

The data supporting this study’s findings are available from the corresponding author upon reasonable request.

## Supplementary Materials

The following supporting information can be downloaded at: https://www.mdpi.com/article/10.3390/ijms242216142/s1.
