# Supplementary material for: Ketogenic Diet Regulates Cardiac Remodeling and Calcium Homeostasis in Diabetic Rat Cardiomyopathy
Source: Int J Mol Sci. 2023 Nov 9;24(22):16142. doi: 10.3390/ijms242216142 (PMC10671812; doi:10.3390/ijms242216142)
Supplement: Supplementary file 1 [file ijms-24-16142-s001.zip › ijms-2664636-supplementary.pdf]

Supplementary Information

Ketogenic diet regulates cardiac remodeling and calcium homeostasis in diabetic cardiomyopathy

Ting-I Lee, Nguyen Ngoc Trang, Ting-Wei Lee , Satoshi Higa, Yu-Hsun Kao, Yao-Chang Chen, Yi-Jen

Supplementary Data

Effects of KD on blood pressure, body weight, and biochemistry of DM rats

Table S1 summarizes the blood pressure (BP), body weight (BW), heart weight (HW), and blood biochemistry in control rats (control), diabetes mellitus (DM) rats on a normal diet (DM+ND), and DM rats on the ketogenic diet (DM+KD). Heart rate (HR), BP, BW, and HW were lower in DM+ND than in control and DM+KD. BW and HW were similar between DM+KD and control. However, HW/BW ratio was higher in DM+ND than in control and DM+KD, whereas it was similar between control and DM+KD.

Table S1. Physical characteristics, blood pressure, and weights DM+ND, and in control, DM+KD.

	Control (N=8)	DM+ND (N=8)	DM+KD (N=8)
**Baseline FBG (mg/dL)**	101.1 ± 12.7	100.8 ± 11.6	102.0 ± 6.1
**FBG (mg/dL)**	100.9 ± 8.5	433.9 ± 33.8[a]	284.3 ± 17.6[a,b]
**SBP (mmHg)**	116.5 ± 5.9	99.0 ± 11.7[a]	110.5 ± 8.8[b]
**DBP (mmHg)**	61.3 ± 10.1	40.8 ± 8.5[a]	52.0 ± 5.7[b]
**HR (bpm)**	426.0 ± 42.8	322.8 ± 48.1[a]	402.0 ± 53.1[b]
**Baseline BW (g)**	365.1 ± 10.7	367.1 ± 9.6	368.8 ± 14.8
**BW (g) after KD**	503.6 ± 29.6	382.8 ± 72.3[a]	422.4 ± 59.6[b]
**HW (g)**	1.79 ± 0.15	1.48 ± 0.21[a]	1.69 ± 0.13[b]
**HW/BW (mg/g)**	3.63 ± 0.3	4.58 ± 0.6[a]	3.87 ± 0.6[b]

Abbreviations: Control: control rats; DM+ND: DM rats on a normal diet; DM+KD: DM rats on ketogenic diet; N: number of rats; FBG: fasting blood glucose; SBP: systolic blood pressure; DBP: diastolic blood pressure; HR: heart rate; bpm: beats per minute; BW: body weight; HW: heart weight. Values are expressed as mean ± standard deviation (SD). Statistical significance was assessed using a one-way analysis of variance (ANOVA) with Tukey's Honest Significant Difference (HSD) test. [a] $P < 0.05$ vs. control; [b] $P < 0.05$ vs. DM+ND.

.

Table S2. Components of Normal Diet and Ketogenic Diet

Normal Diet (ND)		Ketogenic diet (KD)	
**5001*** (Rodent, LabDiet, St Louis, MO, USA)		**5TJR, Rodents,** Test Diet, St. Louis, MO, USA)	
**Protein (%)**	**24.1**	**Protein (%)**	**43**
Arginine (%)	1.51	Arginine (%)	1.68
Cystine (%)	0.38	Histidine (%)	1.24
Glycine (%)	1.24	Isoleucine (%)	2.30
Histidine (%)	0.60	Leucine (%)	4.16
Isoleucine %	1.03	Lysine (%)	3.50
Leucine (%)	1.83	Methionine (%)	1.24
Lysine (%)	1.43	Cystine (%)	0.90
Methionine (%)	0.60	Phenylalanine (%)	2.30
Phenylalanine (%)	1.06	Tyrosine (%)	2.43
Tyrosine (%)	0.74	Threonine (%)	1.86
Threonine (%)	0.94	Tryptophan (%)	0.53
Tryptophan (%)	0.27	Valine (%)	2.74
Valine (%)	1.12	Alanine (%)	1.33
Serine (%)	1.13	Aspartic Acid (%)	3.10
Aspartic Acid (%)	2.71	Glutamic Acid (%)	9.83
Glutamic Acid (%)	4.54	Glycine (%)	0.93
Alanine (%)	1.42	Proline (%)	5.67
Proline (%)	1.43	Serine (%)	2.66
Taurine (%)	0.03	Taurine (%)	0.00
**Fat (ether extract) (%)**	**5.1**	**Fat, %**	**38.41**
**Fat (acid hydrolysis) (%)**	**6.4**	Cholesterol (ppm)	2,164
Cholesterol (ppm)	196	Linoleic Acid (%)	3.30
Linoleic Acid (%)	1.25	Linolenic Acid (%)	0.31
Linolenic Acid (%)	0.12	Arachidonic Acid (%)	0.04
Arachidonic Acid (%)	0.02	Omega-3 FAs (%)	0.44
Omega-3 FAs (%)	0.31	Total Saturated FAs (%)	15.44
Total Saturated FAs (%)	1.39	Total Monounsaturated FAs (%)	12.24
Total Monounsaturated FAs (%)	1.52	Polyunsaturated FAs (%)	6.11
**Fiber (Crude) (%)**	5.3	**Fiber (max) (%)**	**7.8**
Neutral Detergent Fiber (%)	16.9		
Acid Detergent Fiber (%)	7.0		
**Nitrogen-Free Extract (by difference) (%)**	**48.1**	**Carbohydrates (%)**	**2.8**
Starch (%)	21.9		
Sucrose (%)	3.25		

Abbreviation: FAs: fatty acids

Full-length blots of Figure 7. Uncropped full scans of Western blots from the corresponding cropped. Bands chosen for statistical analysis are labeled with the group name. Red rectangle highlights the representative bands. Molecular weight markers are labeled.

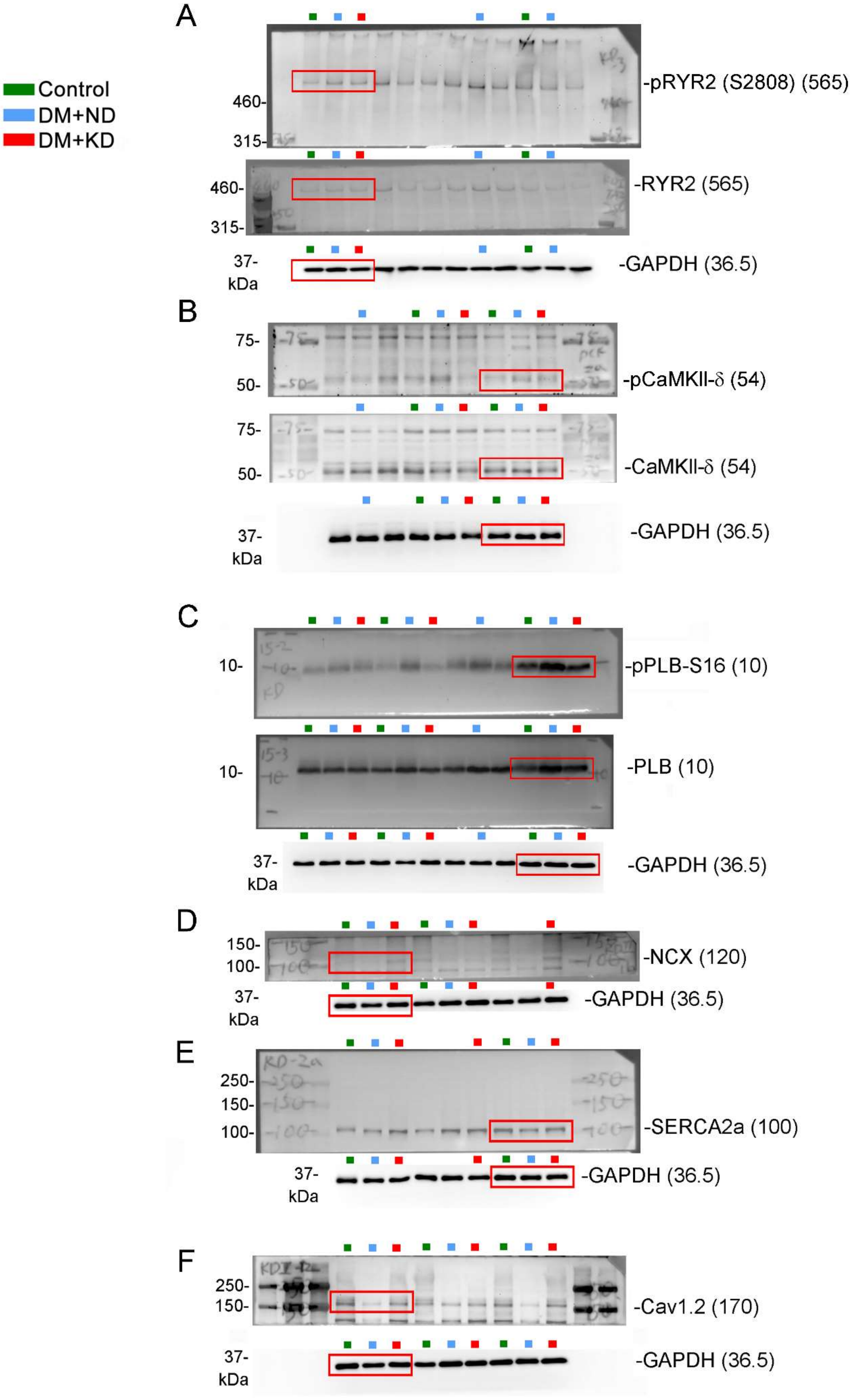
Control
DM+ND
DM+KD
A
460-
315-
-pRYR2 (S2808) (565)
460-
315-
-RYR2 (565)
37-
kDa
-GAPDH (36.5)
B
75-
50-
-pCaMKII-δ (54)
75-
50-
-CaMKII-δ (54)
37-
kDa
-GAPDH (36.5)
C
10-
-pPLB-S16 (10)
10-
-PLB (10)
37-
kDa
-GAPDH (36.5)
D
150-
100-
-NCX (120)
37-
kDa
-GAPDH (36.5)
E
250-
150-
100-
-SERCA2a (100)
37-
kDa
-GAPDH (36.5)
F
250-
150-
-Cav1.2 (170)
37-
kDa
-GAPDH (36.5)
